# Temperature and photoperiod stress in zebrafish larvae: impacts on development, gene regulation and PGC migration

**DOI:** 10.1007/s10695-025-01568-x

**Published:** 2025-09-03

**Authors:** María Santos-Villadangos, Alba Sellés-Egea, Vanesa Robles, David G. Valcarce

**Affiliations:** Cell Biology Area, Molecular Biology Department, Universidad de León, Campus de Vegazana S/N, 24071 León, Spain

**Keywords:** Zebrafish, Organogenesis, Stress, Temperature, Photoperiod

## Abstract

Early life stress is key in development and behaviour and may have long-term effects, as it represents a window of increased vulnerability. In our study we evaluated two stressors, temperature and photoperiod —alone and combined—, in zebrafish (*Danio rerio*) larvae up to 120 h post-fertilisation. The objective of this study was to investigate the effects of non-lethal environmental stressors and their potential synergistic effects until completion of organogenesis integrating molecular, cellular and behavioural analyses. Larvae were subjected to 4 experimental conditions: “control” (C) (14 h light: 10 h darkness; *T* = 27 ± 1 °C); “heat shock” (HS) (14 h light: 10 h darkness; *T* = 34 °C); “darkness” (D) (24 h darkness; *T* = 27 ± 1 °C) and “stressed” (S^+^) (24 h darkness; *T* = 34 °C). Our results confirmed phenotypic, behavioural and molecular alterations. The S^+^ larvae showed a lower survival curve, lower regeneration, a higher number of malformations, predominantly those related to the swim bladder, lower motor activity and a dysregulation of *miR29a* and *clocka*, *hspa9*, *hspa5* and *cxcr4b* genes. The results revealed a similar number of Ddx4^+^ cells between groups but non-canonical migration patterns in the S^+^ group. This integrated approach offers new insights into the molecular mechanisms underlying the stress response during embryogenesis and provides a new perspective on the interaction between temperature and light on early-stage development.

## Introduction

Stress can be defined as a physiological response of the organism that occurs in an attempt to restore homeostasis after its disruption (Tort 2011; Lu et al. 2021). This response is usually non-specific and it depends on the magnitude, duration, type and severity of the stressor (Schreck and Tort 2016; Agorastos and Chrousos 2022). In terms of the duration of the physiological consequences on the organism, stress can be classified into acute or chronic. Acute stress refers to a short-term, physiological and behavioural response to a sudden, often threatening, stimulus, like being caught in a net in the case of a fish. Chronic stress, on the other hand, is a prolonged and persistent stress response to ongoing, often less intense, but inescapable stressors, such as poor water quality or overcrowding (Schreck and Tort 2016). Transitory stressful situations of mild stress (eustress) can have positive adaptive effects on the organism while more severe stress exposure (distress) has negative consequences on the individual (Schreck and Tort 2016; Agorastos and Chrousos 2022). Acute stress involves mild stressors, mild physiological consequences and, usually, homeostasis recovery within a short period of time through stressor compensation. However, under chronic stress conditions, the source of stress is severe, and the response must be prolonged over time, in which the organism may not recover homeostasis (Chu et al. 2024). Thus, this second type of stress has long-term negative consequences, affecting vital functions. In particular, early life stress (ELS) plays a fundamental role in the future development of the organism, potentially affecting growth (Shin et al. 2023), development (Vindas et al. 2018; Nakama et al. 2023), disease resistance (Robinson et al. 2019), behaviour (Eachus et al. 2021) and reproduction (Villamizar et al. 2012; Thumfart et al. 2022). In consequence, during recent years, there has been a growing interest in the scientific community on this topic. ELS, as long-term impact of early life stress, has been demonstrated in humans (Ochi and Dwivedi 2023; Pappalardo et al. 2023) and both mammal (Tao et al. 2023; Muir et al. 2023) and fish (Singh et al. 2022; Fontana et al. 2023) model species and fish with commercial interest (Butzge et al. 2021; Uren Webster et al. 2021).

Focusing on fish, freshwater and marine ecosystems are usually under continuous exposure to multiple stressors, such as temperature variation, contaminants, acidification or hypoxia, among many others (Schreck and Tort 2016). Aquaculture facilities are not an exception, and the presence of stressors can affect the fish productivity leading to economic losses. In the present work, we evaluate two chronic stressors: temperature and photoperiod constantly applied during the whole process of organogenesis. We have selected these crucial stressors for our study, because of their remarkable effect on fish homeostasis (He et al. 2014; Yin et al. 2023), survival rate (Melendez and Mueller 2021) and growth (Strand et al. 2018; Tien et al. 2024) in production facilities.

Temperature limits the speed of cellular biochemical reactions and plays a crucial role in the organism, influencing metabolism, development, growth, reproduction and behaviour (López-Olmeda and Sánchez-Vázquez 2011; Haesemeyer 2020). Temperature alteration outside the tolerance values for each species is a source of stress, leading to negative effects in terms of development, such as malformations, or even death (Pype et al. 2015; Ern et al. 2023). Challenging temperatures for each species also influence fish sensitivity to other stressors like xenobiotics (Osterauer and Köhler 2008; Sanahuja et al. 2020) or hypoxia (Long et al. 2015; McArley et al. 2020). Furthermore, high temperatures have been proven to induce female-to-male sex reversal (Uchida et al. 2004; Han et al. 2021) compromising stock control.

On the other hand, photoperiod directly impacts circadian rhythms, which are endogenous oscillations of 24 h that regulate the physiology and behaviour of the organism and play a fundamental role on maintaining immune system, nervous system, metabolic pathways and cell cycle (Jagannath et al. 2017; Sacksteder and Kimmey 2022). This biological clock is regulated by external synchronising environmental signals called zeitgebers, mainly the exposure to sunlight (Jagannath et al. 2017). Several studies have reported the effect of aberrant photoperiod in survival, which conditions of constant darkness decrease the immune response and therefore increase mortality (Du et al. 2017), growth, where larvae maintained at a 24 h darkness photoperiod showed lower growth (Villamizar et al. 2014) and behaviour, affecting larval activity and swimming pattern (Sorokin et al. 2022).

In the present work, we use *Danio rerio* as a model species in aquaculture to study the impact of chronic stress on early life. Zebrafish have a stress response system that is highly conserved with mammals, as the response is mediated by the hypothalamic-pituitary-interrenal (HPI) axis, which is functionally and structurally homologous to the hypothalamic–pituitary–adrenal (HPA) axis of mammals (Alsop and Vijayan 2009; Eachus et al. 2021). In particular, zebrafish possess a strong and well-defined behavioural and physiological stress response given that this species is highly sensitive to acute and chronic stress (Demin et al. 2021).

While previous studies have demonstrated the impact of temperature and photoperiod on fish physiology and stress response our research aims to provide novel insights by focusing specifically on the early stages of development, particularly the first 120 h post-fertilisation (hpf) and to investigate the immediate effects of single-stress condition —constant darkness and hyperthermia— or their combination during the critical period of embryogenesis. To this end, we employed three environmentally relevant stress scenarios: one involving elevated temperature (34 °C) under a standard light/dark cycle (14 : 10), the second one involving constant darkness and the third one combining both stressors. We aim that these artificial, non-lethal models allow us to dissect the developmental consequences of early environmental disruption in a reproducible way. We hypothesise that altered photoperiod and elevated temperature act as distinct early-life environmental stressors, each capable of inducing specific developmental effects. Moreover, when combined, these stressors may interact synergistically, leading to unique or amplified physiological, molecular and behavioural responses. Studying these stressors both separately and together can help us better understand how complex environmental conditions influence development. It is important to note that the environmental conditions tested in this study do not aim to replicate typical aquaculture scenarios. Instead, we have deliberately modified temperature and photoperiod parameters —two key factors in fish farming— to induce a controlled and non-lethal stress model. By using these relevant environmental variables as stressors, we aim to investigate the physiological and developmental consequences of chronic stress during early stages. This experimental approach provides a reproducible framework for understanding stress responses, with potential applications in the design of management strategies or the identification of early biomarkers for use in aquaculture and related research.

## Materials and methods

### Ethics

All experiments were conducted according to the standard animal guidelines approved by the Bioethics Committee of the University of León (70/6693) and were performed in accordance with Spanish (RD 53/2013) and European legislation (European directive 2010/63/EU). Protocols were approved by the Bioethics Committee of the University of León (70/6693), Junta de Castilla y León (project code: ULE009-2020).

### Animal maintenance

Wild type adult zebrafish (*Danio rerio*; AB line) used in this work as breeders were kept in housing tanks in a recirculating water system under standard conditions with a room photoperiod of 14 h light : 10 h darkness at 27 ± 1 °C at the facilities of the Animal Research and Welfare Service of the University of León (Spain). Progenies were obtained from routine crossings (ratio 1 ♂ : 2 ♀) following standard protocols. Collected embryos from each crossing were kept in embryo medium (EM) (0.137 M NaCl; 5.4 mM KCl; 0.25 mM Na_2_HPO_4_; 0.44 mM KH_2_PO_4_; 6.5 mM CaCl_2_; 4.99 mM MgSO_4_−7H_2_O: 4.2 mM NaHCO_3_; 50 µL 1% (w/v) methylene blue/L EM) (Westerfield 2000) in Petri dishes (Ø = 90 mm) until splitting into experimental replicates.

### Experimental design

**Fig. 1:**
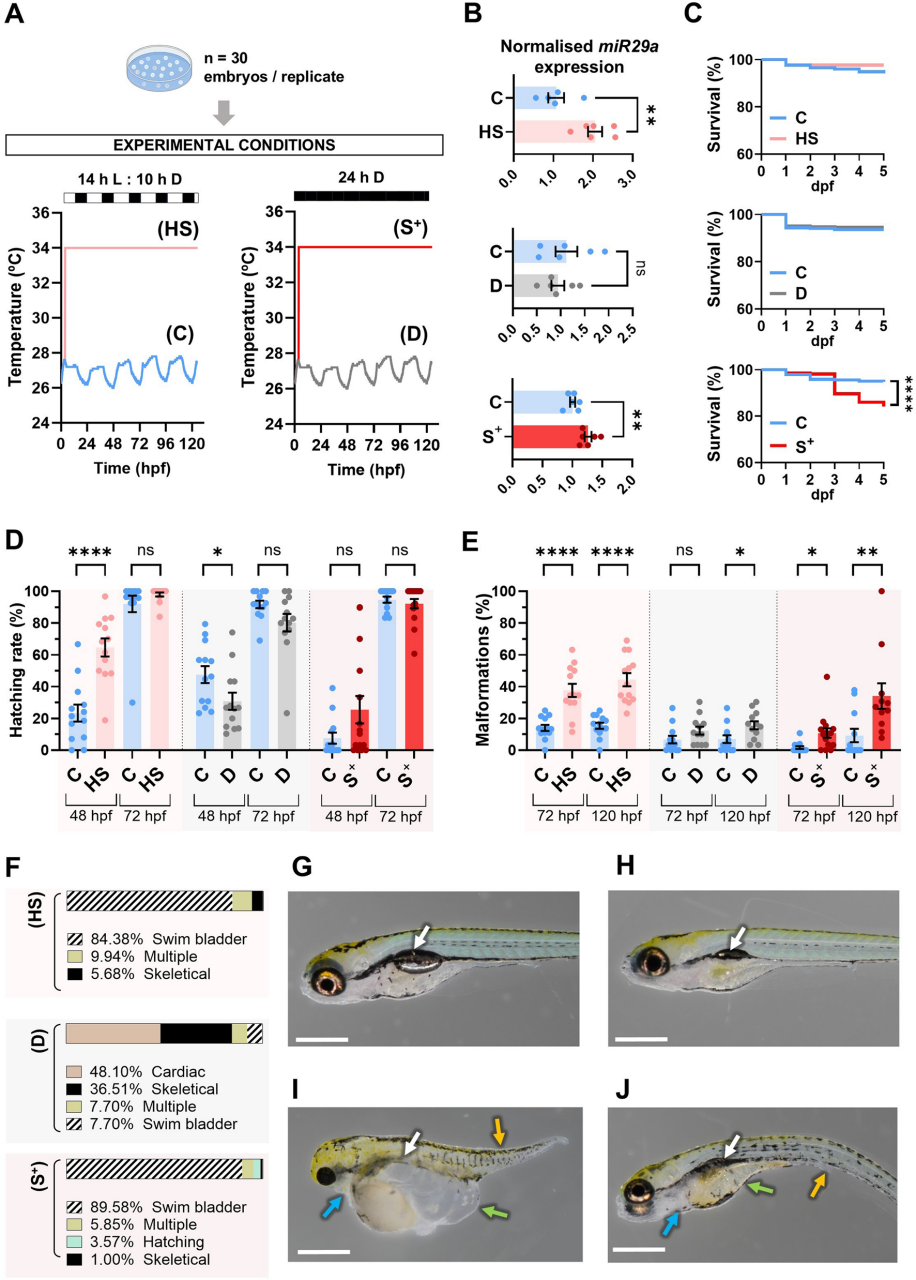
Experimental design and progeny outcome of the experiment. **A** Experimental design. **B** Normalised *mirR29a* expression. Mean value ± SEM is represented (*n* = 6 larvae pools). **C** Kaplan–Meier survival curves (Mantel-Cox test) of the progenies during the experiment. **D** Hatching rate (%) at 48 hpf and 72 hpf. **E** Malformation rate (%) at 72 hpf and 120 hpf. **F** Types of malformations (%) found in larvae at 120 hpf exposed to the challenging culture conditions. **G** Example of a “control” larva at 120 hpf showing a canonical phenotype at this stage. Examples of malformed larvae from the “stressed” (S^+^ ) experimental group at 120 hpf: (**H**) malformed swim bladder, (**I**) and (**J**) severe phenotypes with multiple types of malformations. In **G**–**J**, white arrows indicate the location of the swim bladder, yellow arrows indicate skeletal malformations, green arrows indicate poor yolk sac reabsorption/yolk sac oedema formation and blue arrows indicate pericardial oedema. Biological replicates (*n* = 30 initial embryos/plate) were obtained from crossings (ratio 1 ♂ : 2 ♀). The temperature curves represented were recorded with a data logger (Testo 175T2). The scale of the images is 0.5 mm. Bars in **B**, **D** and **E** show mean value ± SEM (“control” (C) *n* = 13, “heat shock” (HS) and “darkness” (D), *n* = 13; “stressed” (S^+^), *n* = 15). hpf: hours post-fertilisation. *p < 0.0500. **p < 0.0100. ****p < 0.0001

The experiment involved the first 120 h post-fertilisation (hpf) covering the organogenesis of the species. Four experimental groups were established based on culture conditions: 1) “control” (C) —specimens maintained under standard conditions (14 h light : 10 h darkness photoperiod; *T* = 27 ± 1 °C); 2) “heat shock” (HS) —(14 h light : 10 h darkness; *T* = 34 °C)—; 3) “darkness” (D) —(24 h darkness photoperiod; *T* = 27 ± 1 °C)—; and 4) “stressed” (S^+^) —specimens maintained under constant darkness and challenging temperature (24 h darkness photoperiod; *T* = 34 °C)— (Fig. 1A).

The biological replicates included viable and canonically developed embryos at 2.75–3 hpf which were selected from the stock Petri dishes derived from each crossing. Each biological replicate consisted of 30 embryos (*n* = 30) per plate (Ø = 5 mm) maintained in 8 mL of EM.

Once generated, the replicates were randomly assigned to each experimental condition. Plates corresponding to the “control” (C) and “darkness” (D) were kept in the zebrafish room of the animal facility (*T* = 27 ± 1 °C; monitored with a data logger Testo 175T2 under standard photoperiod) exposed or not to light respectively, while those corresponding to the “heat shock” (HS) and “stressed” (S^+^) groups were placed in a Memmert INE 400 incubator programmed at 34 °C under a 14 h light : 10 h darkness or complete darkness, respectively.

### Progeny evaluation

#### Survival, hatching and malformations evaluation

Survival (% number of live specimens/initial number of specimens on the plate) was studied during the 120 h of the experiment. Dead individuals were removed from the plate daily.

At 48 and 72 hpf, hatched eggs were quantified. The hatching rate was calculated in relation to the number of live specimens at the assessment points (% number of hatched specimens/number of live specimens on the assessment day).

The malformation rate (% number of malformed specimens/number of live specimens on the assessment day) and the type of malformations were evaluated at 72 hpf and 120 hpf, concurring respectively with two key points in zebrafish development: end of embryogenesis and end of organogenesis. To illustrate the malformations, extra plates were prepared from each experimental condition to take photographs. The equipment used for this purpose was a Nikon SMZ25 stereomicroscope (camera: Nikon DS-Ri2). NIS-Elements software (Nikon) was used for image processing. The biological replicates used for image acquisition were not included in other analyses to avoid biases in the results.

#### Behavioural analysis

Specimens were recorded at 24 hpf to study embryo behaviour in terms of tail burst movement. Plates (*n* = 7; pool of live embryos per plate at 24 hpf per replicate) were selected from each experimental condition and 1-min videos were recorded under a Nikon SMZ25 stereomicroscope (Nikon DS—Ri2 camera). Each video included all the live embryos within the biological replicate. The resulting videos were used to assess the number of burst movements inside the chorion per individual per minute.

At 120 hpf, collective 10-min zenithal recordings of the live larvae per plate at this keypoint were videotaped on their own plate (*n* = 7), as subjective differences in fish behaviour were observed during the trials. To quantify this observation, plates were randomly selected from each experimental condition for recordings. Plates from the “stressed” (S^+^) and “darkness” (D) groups had a 30 min adaptation period to the light in the zebrafish room before the experiment. Larval activity was calculated by quantifying the percentage of active specimens during the 10 min/number of live specimens on the day of evaluation.

### Molecular studies

#### Sample collection

At 120 hpf (between 09:00 h and 10:00 h right after behavioural recordings were finished), the embryo medium was removed from 6 plates from each experimental condition and their control counterparts were euthanised with a lethal dose of tricaine (MS222—0.015 M) following standard protocols (Westerfield 2000). Subsequently, the tricaine solution was removed and washed with PBS 1 × (0.8% NaCl; 0.02% KCl; 0.02 M PO_4_). Larval pools from each plate (*n* = 6/experimental condition) were placed in labelled 1.5 mL tubes containing 250 µL of RNA later (Invitrogen) and stored at − 80 °C until processing. These samples were used for two purposes: 1) a molecular-level certification of stress induction by an alteration of the expression of a previously described (Riesco et al*. *2023); Valcarce et al. 2024) stress-related miRNA, *miR29a*, and 2) an exploration of the impact of the experimental conditions on the expression of candidate genes.

#### RNA extraction

RNA extraction was performed with the miRNeasy Tissue/Cells Advanced Mini Kit (Qiagen) following the protocol described by the commercial company. Quantification and purity of extracted RNA was determined with a DS-11 spectrophotometer/fluorometer (DeNovix). Samples showed absorbance indices A260/A280 and A230/280 ≥ 1.8–2.0.

#### Retrotranscription

##### RT for miRNA

For each RNA sample, we performed retrotranscription reactions (10 ng of total starting RNA) using the TaqMan MicroRNA Reverse Transcription Kit (Applied Biosystems) with specific TaqMan Small RNA probes (5 ×) for *miR29a* (MIMAT0001802) and *miR92a-3p* (MIMAT0001808), following manufacturer’s protocol. *miR92a-3p* was selected as reference miRNA as previously reported (Riesco et al. 2019; Valcarce et al. 2024).

##### RT for mRNA

For each RNA sample, we performed retrotranscription reactions (1 µg of total starting RNA) using the High-Capacity RNA to cDNA kit (Applied Biosystems) following manufacturer’s protocol.

#### Quantitative PCR

##### qPCR for miRNA

qPCR for miRNA was performed under standard cycling conditions following manufacturer’s guidelines. The composition of each reaction (20 µL) was 10 µL of TaqMan Fast Advanced Master Mix (2 ×), 7.67 µL molecular biology degree H_2_O, 1 µL of TaqMan MicroRNA Assay (20 ×) (*miR29a* and *miR92a-3p*) and 1.33 µL cDNA (2 ng/µL). Three technical replicates were performed for each sample and gene on MicroAmp Optical 96-Well Reaction Plates (Applied Biosystems) in a Quant Studio 1 thermal cycler (Applied Biosystems).

##### qPCR for selected genes

The genes selected for the qPCR study were *clocka* (clock circadian regulator a), *bmal1b* (basic helix-loop-helix ARNT like 1b), *casp3* (caspase 3, apoptosis-related cysteine peptidase), *hspa5* (heat shock protein family A member 5), *ddit3* (DNA damage inducible transcript 3), *hspa9* (heat shock protein family A member 9), *sox2* (SRY-box transcription factor 2), *rarga* (retinoic acid receptor gamma a), *ddx4* (DEAD-box helicase 4), *cxcl12a* (C-X-C motif chemokine ligand 12a) and *cxcr4b* (C-X-C motif chemokine receptor 4b). The composition of each reaction (20 µL) was 10 µL of SYBR Green PCR Master Mix (Applied Biosystems), 2 µL of primers (500 nM; forward + reverse) (Table 1), 0.5 µL cDNA (25 ng/µL), and 7.5 µL molecular biology degree H_2_O. Three technical replicates were performed for each sample and gene on MicroAmp Optical 96-Well Reaction Plates (Applied Biosystems) in a Quant Studio 1 thermal cycler (Applied Biosystems). The qPCR cycling conditions were standard and included a melting curve study for each gene studied. The reaction products were also run on a 1.5% agarose gel to confirm the absence of non-specific bands.

**Table 1:** Forward and reverse primers used for qPCR

Gene	Accession number	Oligo	Secuence (5′ to 3′)	Amplicon size (bp)	Melting temperature (°C)	Ref
*actb2*	*NM_181601.5*	F	CGTGCTGTCTTCCCATCCA	86	60	Riesco and Robles (2013)
		R	TCACCAACGTAGCTGTCTTTCTG			
*cxcr4b*	*NM_131834.1*	F	GGCGCTGGCATATTTCCA	57	62	
		R	ACGCCTAGGAAAGCATAAAGGA			
*eef1a1l1*	*NM_131263.1*	F	CTGGAGGCCAGCTCAAACAT	87	60	Divisato et al. (2016)
		R	ATCAAGAAGAGTAGTACCGCTAGCATTAC			
*cxcl12a*	*NM_178307.2*	F	ATTCGCGAGCTCAAGTTCCT	214	62	Lombó et al. (2019)
		R	ATATCTGTGACGGTGGGCTG			
*casp3*	*NM_131877.3*	F	GGCAGATTTCCTCTATGCATACTC	72	60	Matsumoto et al. (2021)
		R	CATGAGCCGGTCATTGTG			
*hspa5*	*NM_213058.1*	F	AAGAGGCCGAAGAGAAGGAC	133	60	
		R	AGCAGCAGAAGCCTCGAAATA			
*ddit3*	*NM_001082825.1*	F	AAGGAAAGTGCAGGAGCTGA	197	60	
		R	TCACGCTCTCCACAAGAAGA			
*ddx4*	*NM_131057.1*	F	ATGGCATTCCCATCATTTCAG	74	62	This work
		R	GGCCGCCGTTTTTCCT			
*hspa9*	*NM_201326.2*	F	CCCCACTGTCTCTTGGCATT	105	60	
		R	CCGCAGTAGAGAACACCTGG			
*sox2*	*NM_213118.1*	F	ACTCCATGACCAACTCGCAG	159	60	
		R	AATGAGACGACGACGTGACC			
*clocka*	*NM_130957.2*	F	CACCAACCTCAACCAGCAGTCT	185	60	
		R	GTGAGAAGGTTGCGACGGC			
*bmal1b*	*NM_178300.1*	F	GTCTCCCAGTTCCACTGAGC	129	60	
		R	GTGTCCATACTGTCCTCAAAAGAA			
*rarga*	*NM_131339.1*	F	CACGAGGCTAGGAACAGCTC	138	60	
		R	ATACGTGCTGCCTGATCCTG			

The normalised expression of each gene was calculated using the Pfaffl’s method (Pfaffl 2001). Two housekeeping genes (HKG) were studied before starting the analysis: β-actin 2 (*actb2*) and eukaryotic translation elongation factor 1 alpha 1 like 1 (*eef1a1l1*). The β-actin 2 was selected as a reference gene using the RefFinder tool (Xie et al. 2012). Accession number, primer sequences, amplicon length (bp) and melting temperature (°C) are presented in Table 1.

#### *Whole mount* immunofluorescence staining

*Whole mount* immunofluorescence staining was performed on control and malformed larvae of the “stressed” (S^+^) group at 120 hpf following previously published protocols (Lombó et al. 2019) with slight modifications. First, larvae were fixed in 4% paraformaldehyde (PFA) in 1 × PBS at 4 °C overnight. The samples were kept in 70% ethanol at 4 °C until the protocol was completed. We conduct two 5-min washes in 1 × PBS, followed by a permeabilisation with methanol at − 20 °C for 2 h. After this time, three 5-min washes were performed in Tris-buffered saline with 1% Triton X-100 (TBS-T 1%). Blocking was then performed with a 3% (w/v) solution of bovine serum albumin (BSA) in 1% TBS-T for 2 h at room temperature. Subsequently, the volume of blocking solution was removed and the samples were incubated with the polyclonal anti-Ddx4 primary antibody (CTX128306, GeneTex) diluted 1:200 in blocking solution for 2 days at 4 °C. After incubation, we perform three 5-min washes in 1% TBS-T and incubate with the secondary antibody (goat anti-rabbit Alexafluor 488) diluted 1:500 in 1% TBS-T at 4 °C overnight. After 3 washes of 5 min in 1 × TBS-T the specimens were analysed under fluorescence.

### Fin regeneration

To evaluate the potential effect of “stressed” (S^+^) conditions on tissue regeneration, we prepared extra plates to study blastema regeneration. At 72 hpf, we amputated the caudal fin of randomly selected larvae of each experimental condition (*n* = 7) following previous published protocols (Pase et al. 2012). The cut was made at the end of the notochord as a reference point. After amputation, larvae were returned to their respective conditions. At 7 dpf, we took photos of the area regenerated using a Nikon SMZ25 stereomicroscope (Nikon DS—Ri2 camera). The software used for image processing and area quantification was NIS-Elements and Adobe Photoshop CS3.

### Statistical analysis

Data were analysed and plotted with GraphPad Prism 8.0.1 software (GraphPad Software). Survival curve comparison was analysed using the Mantel-Cox test. To explore the normality of the variables, a Shapiro–Wilk test was performed. When two normal variables were compared, a Student’s *t*-test or Welch’s *t*-test was used, while non-parametric variables were compared using a Mann–Whitney test. The *n* corresponding to the number of biological replicates is described in each figure caption. Error bars in the graphs represent means ± standard error of the mean (SEM). *P* values < 0.0500 were considered statistically significant.

## Results

### Molecular-level certification of stress induction

The *miR29a* expression reported statistically significant differences between the “control” group and the “heat shock” (*p* = 0.0050) and “stressed” (*p* = 0.0046) ones, showing an overexpression in the larvae exposed to hyperthermia (HS, 2.050 ± 0.1767; S^+^, 1.262 ± 0.0566) in contrast to the “darkness” larvae, which did not show differences against control-cultured fish (Fig. 1B).

### Progeny evaluation

#### Survival, hatching and malformations evaluation

The survival curve showed no differences between “control” and “heat shock” or “darkness” groups (Fig. 1C), whereas statistically significant differences were reported between “control” (C) and “stressed” (S^+^) larvae (*p* < 0.0001), with a lower survival rate in the S^+^ condition (Fig. 1C).

In terms of hatching, at 48 hpf, the number of hatched larvae were significantly lower in the “darkness” (D) (*p* = 0.0372) and higher in the “heat shock” (HS) (*p* < 0.0001) groups compared to control specimens, while no differences were reported in the “stressed” (S^+^) group (Fig. 1D—48 hpf). However, any statistically significant differences were observed between the conditions at 72 hpf (Fig. 1D—72 hpf). All experimental groups showed hatching rates above 90% (Fig. 1D—72 hpf).

At 72 hpf, statistically significant differences in malformation rate (%) were observed between “control” (C) larvae and challenging conditions involving hyperthermia (HS, *p* < 0.0001; S^+^, *p* = 0.0018 Fig. 1E). The “darkness” (D) trial did not show differences in malformation ratio at 48 hpf (Fig. 1E). At 120 hpf, we registered statistically significant differences between all experimental groups comparing to their control counterparts (HS, *p* < 0.0001; D, *p* = 0.0175; “S^+^”, *p* = 0.0034. Figure 1E). The “heat shock” plates showed a malformation rate mean percentage of 44.42 ± 4.136%, the “darkness” ones a 15.71 ± 2.558%, and the “stressed” plates a mean value of 34.07 ± 8.082%, approximately three times higher than the “control” groups in the hyperthermia trials (Fig. 1E). The type of malformations found in the S^+^ and HS groups were malformations affecting the development of the swim bladder (absence or very reduced volume), being this phenotype predominant with 84.38% in HS and 89.58% in S^+^ groups of total malformed larvae, followed by severe phenotypes involving multiple malformations (9.94% in HS and 5.85% in S^+^), hatching malformations (3.57% in S^+^) and skeletal malformations (5.68% in HS and 1.00% in S^+^) (Fig. 1F). Darkness plates showed different percentages of types of malformations, being cardiac malformations the most abundant (48.10%) followed by skeletical ones (36.51%) (Fig. 1F). Figure 1G–J show representative images of the main phenotypes found in the present experiment.

#### Behavioural analysis

**Fig. 2:**
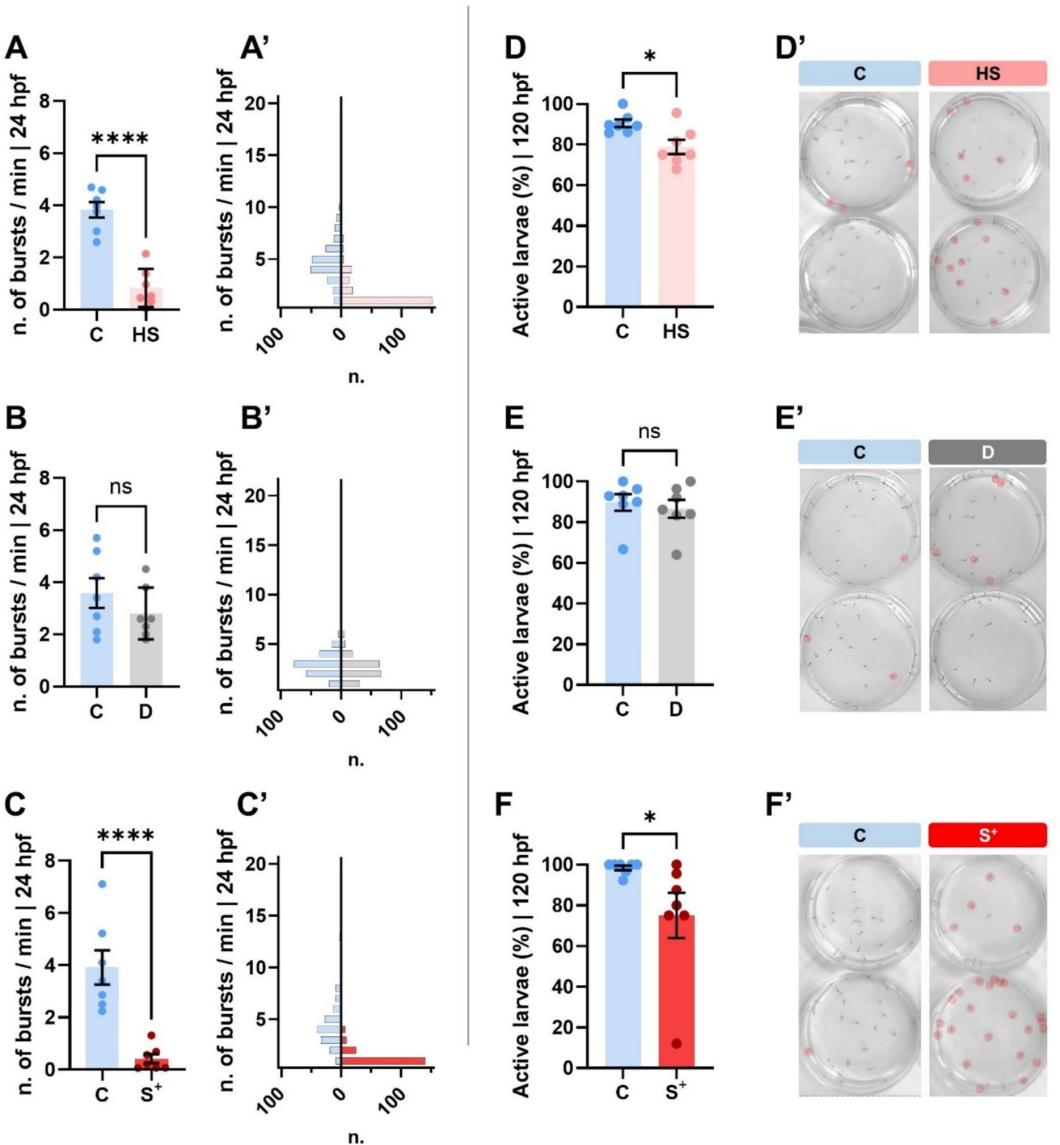
Behavioural analysis. Number of embryo bursts at 24 hpf within the chorion for the “heat shock” (HS) (**A**), “darkness” (D) (**B**) and “stressed” (S^+^) trials (**C**). Frequency distribution histograms of the number of movements per minute between “control” (C) and experimental conditions (**A’–C’**). Active larvae at 120 hpf (%) for the “heat shock” (HS) (**D**), “darkness” (D) (**E**) and “stressed” (S^+^) trials (**F**). **D’–F’** Captures of the videos showing immobile larvae (red circles) at 120 hpf during the behavioural analysis. Bars mean value ± SEM (*n* = 7 larvae pools). *p < 0.0500. ****p < 0.0001

We registered statistically significant differences in the number of bursts per minute of larvae inside the chorion at 24 hpf between the “control” (C) groups and the “heat shock” (HS) and “stressed” (S^+^) conditions (*p* < 0.0001) while “darkness” (D) embryos reported similar mean values (Fig. 2A–C). The frequency distribution of the number of movements per minute showed a mean percentage around 4% in all the “control” groups while the “heat shock” and “stressed” plates showed mean percentages 0.8276 ± 0.2750% and 0.4229 ± 0.1758% respectively (Fig. 2A and C).

At 120 hpf, our behaviour experiment reported a lower activity (Fig. 2D and F) of the both hyperthermia-exposed larvae “HS” (*p* = 0.0130) and “S^+^” (*p* = 0.0111), showing a higher number of individuals that did not perform any movement in the plate during the 10-min video recording (highlighted with red circles in the captures included in Fig. 2D’–F’; clips in Online Resource 1). This observation was not reported in the “darkness” (D) trial (Fig. 2E), which showed similar values to control larvae. The “control” (C) replicates showed mean values close to 90% of motile larvae, while in the replicates of the “heat shock” (HS) and “stressed” (S^+^) groups the mean values were respectively reduced to 78.89 ± 3.523% and 75.02 ± 11.13% (Fig. 2D and F).

### Molecular studies

#### Gene expression evaluation by qPCR

**Fig. 3:**
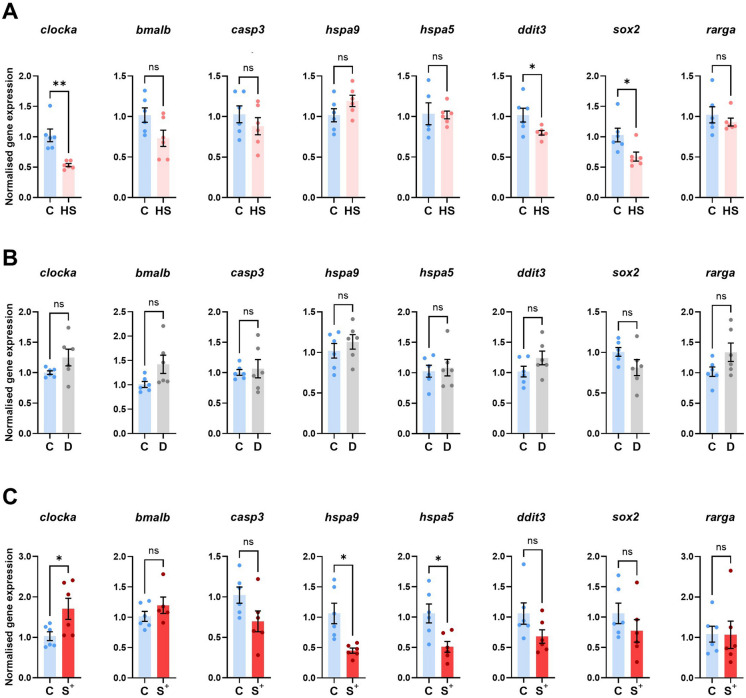
Normalised gene expression (HKG: *actb2*) of 120 hpf larvae (pool of 25 specimens/replicate) for the studied candidate genes (*clocka, bmal1b*, *casp3*, *hspa9*, *hspa5*, *ddit3*, *sox2* and *rarga*) in each trial: “heat shock” (HS) (**A**), “darkness” (D) (**B**) and “stressed” (S^+^) (**C**). Mean value ± SEM (*n* = 6) is represented. *p < 0.0500. **p < 0.0100. “ns” not statistically significant (*p* > 0.0500)

Gene expression analysis only reported statistically significant differences in two of the studied potentially stressful experimental conditions: “heat shock” (HS) and “stressed” (S^+^). Unexpectedly, the “darkness” (D) group did not report any differences in the candidate genes studied here (Fig. 3B).

The gene expression of the two circadian rhythm-related genes, *clocka* and *bmal1b*, showed statistically significant differences in the first one in both plates exposed to hyperthermia (Fig. 3A and C). However, the pattern was different between both experimental conditions. While HS replicates showed a downregulation of *clocka* (*p* = 0.0011), S^+^ reported a notable overexpression (*p* = 0.0383; 1.705 ± 0.2616) in contrast to the C larvae (1.030 ± 0.1081) (Fig. 3A and C). The other studied circadian-cycle gene did not report any statistically significant differences in any of our qPCR experiments (Fig. 3A–C).

Focusing on the studied apoptosis-related genes, *casp3* and *hspa9*, we only found statistically significant differences in the experimental group exposed to both stressors (S^+^) in *hspa9* (*p* = 0.0054). This gene showed downregulation (0.45 ± 0.04211) in the S^+^ group compared to the standard cultured larvae (1.063 ± 0.1683) (Fig. 3C). In contrast, *casp3* showed no statistically significant differences between groups (Fig. 3C).

We studied the expression of other two genes also related to apoptosis and endoplasmic reticulum (ER) stress: *hspa5* and *ddit3*. Again, only the “stressed” (S^+^) larvae reported statistically significant differences, in this case for *hspa5* gene, which showed a statistically significant (*p* = 0.0118) downregulation (0.5100 ± 0.0886) (Fig. 3C). *ddit3* gene expression did not report statistically significant differences in the most challenging experimental group (S^+^) although the normalised gene expression showed a similar trend to *hspa5* in this group (Fig. 3C). We also found no differences for these two genes in the group subjected to heat shock (HS) (Fig. 3A).

On the contrary, the expression of *sox2*, which is a transcription factor with a key role in early development, only showed statistically significant differences (*p* = 0.0260) in the “heat shock” (HS) larvae (Fig. 3A), while the expression of this gene remained similar to the control replicates in the S^+^ group (Fig. 3C). Retinoic acid receptor γ, *rarga*, acid receptor γ and a key gene in development, did not show differences between conditions (Fig. 3).

In relation to the genes that showed significant differences at 5 dpf in the S^ +^, we evaluated them in three-month-old individuals. However, no significant differences were found between the experimental groups (Online Resource 2).

### Evaluation of the number of primordial cells and their migration

**Fig. 4:**
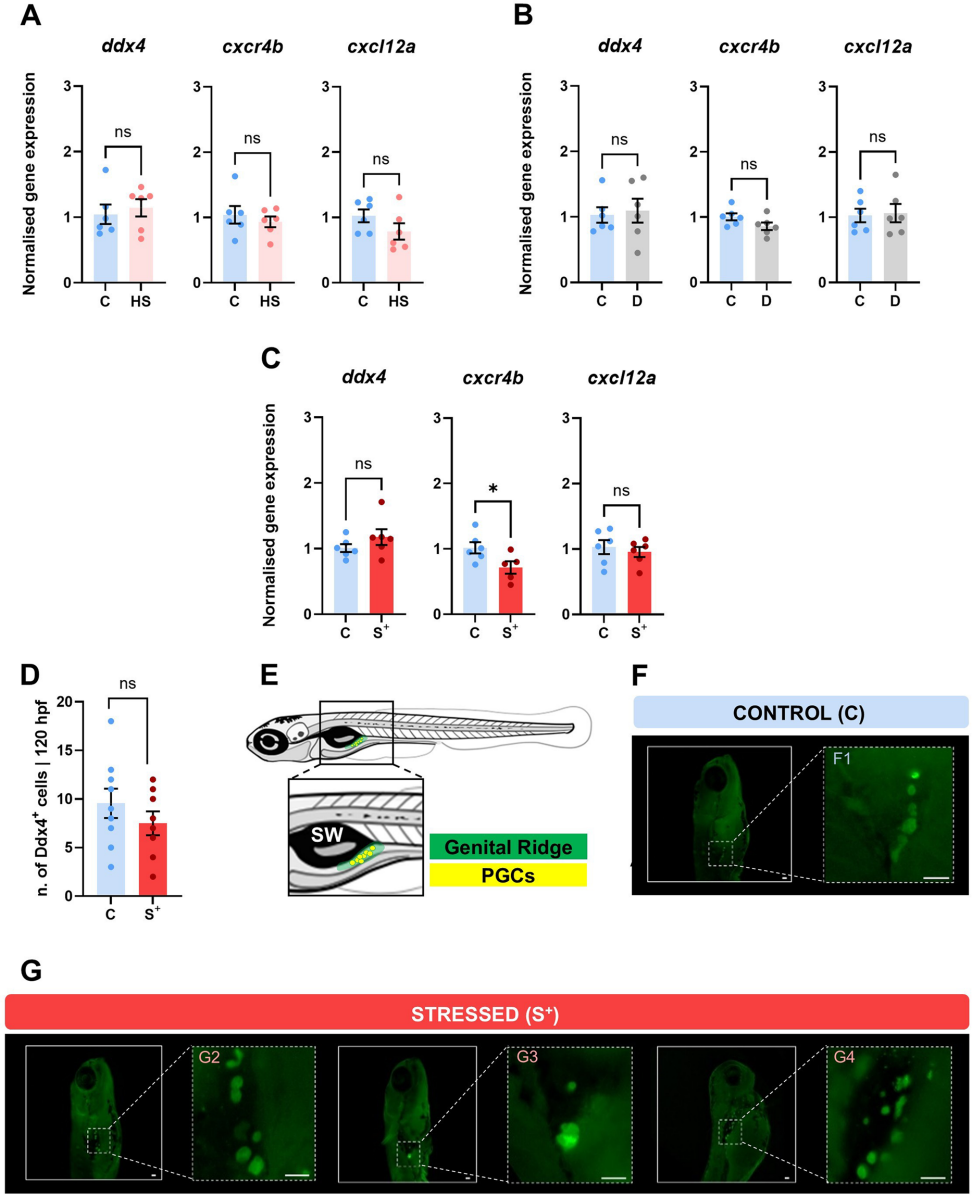
Primordial germ cells (PGCs) analysis. Normalised gene expression (HKG: *actb2*) of genes related to PGCs and their migration to the genital ridge (*ddx4*, *cxcr4b* and *cxcl12a*) in “heat shock” (HS) (**A**), “darkness” (D) (**B**) and “stressed” (S^+^) 120 hpf larvae (**C**). **D** Number of Ddx4^+^ cells at 120 hpf “stressed” (S^+^) larvae. **E** Diagram of a 120 hpf larva showing the genital ridge location and the expected PGC niche at this developmental stage. SW refers to swim bladder. Migration patterns of Ddx4^+^ cells at 120 hpf larvae: **F**) example of a “control” (C) larva showing a canonical ordered distribution pattern (F1) and **G**) examples of “stressed” (S^+^) larvae showing a loose distribution of cells (G2 and G4) and a high concentration of cells in a spot (G3). Mean ± SEM (*n* = 6; pool of 25 specimens/replicate) is represented. *p < 0.0500. “ns” not statistically significant (*p* > 0.0500)

Gene expression analysis of *ddx4* and *cxcl12a* showed no statistically significant differences in any of the experimental groups (Fig. 4A–C). Only *cxcr4b* showed statistically significant differences (*p* = 0.0427), being downregulated (0.7160 ± 0.09411) in the “stressed” (S^+^) group (Fig. 4C). No significant differences were found in the number of Ddx4^+^ cells present in “control” (C) and “stressed” (S^+^) larvae (*p* = 0.3149) (Fig. 4D). Focusing on the PGC migration ability to the genital ridge (Fig. 4E), immunofluorescence analysis reported different migration patterns of Ddx4^+^ cells between “control” (C) and “stressed” (S^+^) groups. While “control” (C) showed an ordered distribution pattern (Fig. 4F), the “stressed” (S^+^) group showed a loose distribution of cells or a high concentration of cells in the genital ridge (Fig. 4G).

### Regeneration capacity evaluation

**Fig. 5:**
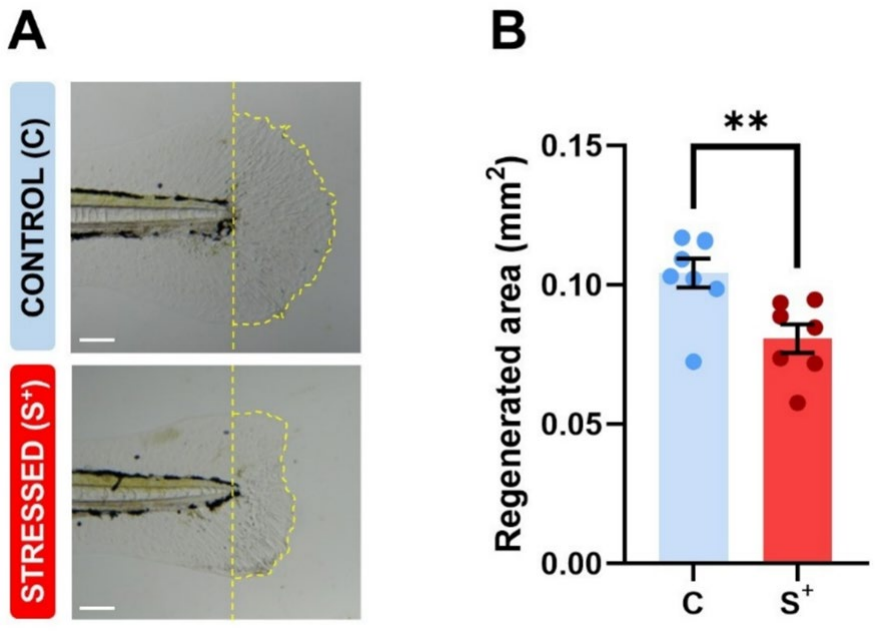
Regeneration capacity of larvae at 7 dpf. **A** Comparison of the regenerated fin area between a “control” (C) and a “stressed” (S^+^) larva. **B** Regenerated fin area (mm^2^). The scale of the images is 0.025 mm. Mean ± SEM (C: *n* = 8; S^+^: *n* = 7) is represented. **p < 0.0100

The blastema area quantification (an example of the regenerated area of each group is illustrated in Fig. 5A) showed statistically significant differences (*p* = 0.0059; Fig. 5B). The “control” group presented a regenerated area mean of 0.1043 ± 0.0052 mm^2^; in contrast with the “stressed” (S^+^) group, which showed a decreased regeneration (0.0807 ± 0.0051 mm^2^).

## Discussion

In this study, we investigated the impact of temperature and photoperiod alterations as stressors during early zebrafish development to gain deeper insights into how environmental factors influence critical early life stages in aquatic organisms. These findings are particularly valuable for aquaculture, where optimising rearing conditions to minimise stress can prevent malformations and reproductive issues, improve survival rates, and ultimately reduce economic losses.

Temperature and photoperiod are critical environmental factors that can act as potent stressors during early developmental stages in aquatic organisms. Deviations from optimal ranges can disrupt molecular pathways and physiological processes, making survival and hatching rates essential indicators of developmental success. To certificate at a molecular level the induction of stress we studied *miR29a* levels. Our group has previously reported a dysregulation of this miRNA in early stages of zebrafish, showing a downregulation in 7 dpf larvae whose male progenitors had been exposed to chronic stress (Riesco et al. 2023) and an overexpression in larvae exposed to an unexpected chronic stress protocol during the first days of life (Valcarce et al. 2024). This second observation is in line with the results registered in this work, which report a *miR29a* overexpression in larvae exposed to both the high-temperature stressor and in the combined condition, but not under constant darkness alone (Fig. 1B). As suggested by this result, the darkness condition did not produce significant changes in the malformation rate at 72 hpf (Fig. 1E), nor in the number of burst movements (Fig. 2B) or the percentage of active larvae (Fig. 2E). Moreover, none of the genes analysed showed significant differences in expression under constant darkness (Fig. 3B).

In terms of global parameters of fish progeny development, our results showed exclusively significant differences in the survival curve between “control” and “stressed” larvae (Fig. 1C). This is supported by several studies (Pype et al. 2015; Hosseini et al. 2019). In the first one, embryos were incubated during 96 hpf at 34.5 °C and 36.5 °C, highlighting the effect of high temperatures on the larvae fate (Pype et al. 2015). The second one evaluated the effects of a heat shock at 35 °C during early development (5–24 hpf), reporting a decrease in the Kaplan–Meier survival curve in the heat shock larvae in comparison with the control ones (Hosseini et al. 2019). Contrary to this previous evidence, our “heat shock” (34 °C) trial did not report statistically significant differences in the survival curve (Fig. 1C), which is particularly interesting as it offers a non-lethal model to investigate stress effects. Attending to hatching ability, we initially expected to register differences at the end of embryogenesis at 72 hpf between our experimental groups in both HS and S^+^ groups since previous publications showed changes in hatching rates under different temperature conditions. Villamizar et al. (2012) reported a higher hatching rate at this temporal keypoint (72 hpf) in larvae maintained at 28 °C compared to those reared at suboptimal conditions (24 °C). Also, and, in line with our HS results (Fig. 1D—48hpf), zebrafish embryos incubated at higher temperatures (30.5 °C, 32.5 °C and 34.5 °C) also showed an increased hatching rate compared to controls (Pype et al. 2015). However, our hatching results at 72 hpf (Fig. 1D) did not show differences between groups, showing values close or equal to 100%. This observation is interesting because although contrary to Pype et al. (2015), where all animals were exposed to the same photoperiod, our experimental stress conditions combine constant darkness and challenging high temperatures.

Photoperiod and circadian rhythm are crucial components in the regulation of various biological and physiological functions in living organisms and their alteration can be an important source of stress. We explored the impact of constant darkness alone and combined with high-temperature stress during organogenesis as a drastic alteration of natural conditions. It has been showed that the alteration of normal light/dark cycles in zebrafish as a model species affects larvae survival and growth, and also that prolonged constant darkness is lethal if maintained further than hatching (Villamizar et al. 2014). Beyond model species, light/dark rhythms and temperature have been also studied (Villamizar et al. 2013) in diurnal, nocturnal and blind fish species, showing that hatching rates were synchronised with photoperiod and temperature. As expected, hatching rates were better under normal photoperiod and optimal temperature for each of the studied species (Villamizar et al. 2013) indicating the importance of adjusting environmental conditions since the beginning of the culture protocol. In our study, the constant darkness stressor alone did not affect hatching at 72 hpf, although at 48 hpf there was a reduction in the number of hatched embryos compared to the control group (Fig. 1D).

The suboptimal rearing conditions may potentially result in malformed individuals. Analysing malformations during early developmental stages in fish is crucial for understanding the impact of environmental stressors on organism health, viability and in terms of production, final product to be sent to the market. Non-canonical phenotypes can serve as visible indicators of underlying physiological and genetic disruptions, often reflecting adverse conditions experienced during development (Noble et al. 2011; Sun et al. 2020; Shahjahan et al. 2022). In fish, these abnormalities can impair essential functions. Moreover, evaluating malformations is essential not only for assessing the immediate effects of stressors on survival and development but also for understanding long-term consequences, such as impaired movement, feeding behaviour and reproductive success. Our study showed a significantly higher rate of malformations in both HS and S^+^ groups at 72 hpf and 120 hpf (Fig. 1E). The main malformations identified were aberrant swim bladder, absence of hatch, skeletal malformations and oedema formation (Fig. 1F–J). Previously, other authors had documented that zebrafish embryos maintained within the range between 25 and 33 °C, appeared to develop canonically, and suggested that prolonged exposure to higher or lower temperatures beyond these limits could lead to developmental abnormalities (Kimmel et al. 1995). Previous studies (Villamizar et al. 2012; Pype et al. 2015) support the results reported here, where the rate of malformations is significantly higher in the challenging culture conditions (Fig. 1E). However, the type of malformations found in our study —being those related to swim bladder the most abundant (Fig. 1F)— differs from the study by Pype et al*.*, who registered mainly cardiovascular and cranioencephalic malformations, oedema and accumulations of blood throughout the body in larvae exposed to hyperthermia under standard photoperiod (Pype et al. 2015). Interestingly, our results show that thermal stress alone is sufficient to induce significant alterations in hatching at 48 hpf (Fig. 1D) and malformation rates (Fig. 1E). However, survival was not notably affected under thermal stress in isolation (Fig. 1C), suggesting that certain developmental endpoints may be more sensitive to specific stressors or their interaction. These findings highlight that increased temperature, even without a synergistic effect with photoperiod disruption, can drive marked developmental disturbances. In the case of embryos exposed to constant darkness, no significant differences in malformation rates were detected at 72 hpf compared to the control group. However, by 120 hpf, a significant increase in malformations became evident (Fig. 1E). Interestingly, the predominant type of malformation observed under constant darkness was cardiac-related, in contrast to the more commonly observed swim bladder defects associated with elevated temperature or combined stress conditions (Fig. 1F). It would be of interest to conduct further molecular studies to explore the potential involvement of genes related to heart morphogenesis that could be disrupted by this specific stressor.

In addition to effects on survival, hatching rates and malformations, stress can substantially modify the behavioural patterns of animals (de Abreu et al. 2021). In the case of fish, behaviour can be studied by analysing their swimming pattern (Martineau and Mourrain 2013; Lucon-Xiccato et al. 2022). However, it should be noted that a differential swimming pattern may also be due to certain malformations in the larvae, especially if these are skeletal malformations or malformations affecting the swim bladder. In the present study, constant darkness did not affect the behavioural parameters assessed, as no significant differences were found in either the number of bursts per minute (Fig. 2B) or the percentage of active larvae (Fig. 2E) but significant differences in movement were observed between the C and HS and S^+^ embryos, which showed a lower number of movements in the chorion at 24 hpf (Fig. 2A and C). These differences were also registered in 120 hpf-larvae (Fig. 2D and F) with reduced activity in larvae cultured under suboptimal conditions. Concomitantly, these 120 hpf-larvae, showed an abnormal vertical swimming pattern, as a consequence of the defects on swim bladder inflation —the highest percentage of malformation type registered—. Several studies relate temperature alterations to behaviour and swimming pattern in *D. rerio* (Haesemeyer et al. 2015, 2018) since larvae movements such as turning or swimming in a straight line, are influenced differently by sensory neurons activated by cold and heat. The impact of photoperiod on zebrafish behaviour is not exclusive to earliest stages of life. Sorokin et al. (2022), compared 6 week-old fish subjected to a short photoperiod (4 h light : 20 h darkness) for 60 days with standard cultured counterparts reporting a decrease in locomotor activity in a Novel Tank Diving Test. In our study, the differences in movement observed between groups at 120 hpf in the S^+^ trial (Fig. 2F) may be due to a synergistic effect between alterations in photoperiod and temperature, as the S^+^ group shows a significant reduction on locomotor activity with the control.

Moving to our molecular exploration, in the present study we analysed 3 groups of genes: 1) those related to circadian rhythm, such as *clocka* and *bmal1b*; 2) those related to apoptosis, such as *hspa9* and *casp3*, and 3) those related to endoplasmic reticulum stress, such as *hspa5* and *ddit3*. Our results have shown significant differences in *clocka*, *hspa9* and *hspa5* in the “stressed” (S^+^) group in relation to the “control” (C) group (Fig. 3C). Photoperiod plays a major role in the maintenance of the circadian rhythm and it is expected that if the photoperiod is altered under conditions of constant darkness, the gene expression of some of the genes related to it will be altered. For example, the study by Basili et al. (2020) showed that constant dark conditions in 72 hpf zebrafish larvae after a normal photoperiod altered the expression of *clock* genes. In addition, circadian rhythm-controlled genes are also involved in lipid metabolism as well as other metabolic pathways, and loss or alteration of their function is associated with the appearance of abnormal metabolic phenotypes (Gooley and Chua 2014), which may be one of the causes of the malformations found in the larvae of the “stressed” (S^+^) group (Fig. 2H–J). Interestingly, *clocka* expression was found to be downregulated in larvae exposed to elevated temperature (Fig. 3A), overexpressed under the combined stress condition (Fig. 3C), and unchanged in constant darkness (Fig. 3B). This result was unexpected, particularly given that alterations in circadian gene expression are commonly associated with disrupted light cycles. A possible explanation is that, in isolation, constant darkness does not generate a sufficiently intense stress signal to impact *clocka* expression at the developmental stage analysed. In contrast, the combined stressor may have triggered compensatory or synergistic mechanisms involving the circadian system, reflected in the observed overexpression. The divergent regulation of *clocka* across conditions highlights the complex interplay between temperature and photoperiod in circadian control during early development. Sánchez-Vázquez et al*.* observed that the expression of *clocka* and *bmal* genes was affected by lighting conditions during early larval development (Sánchez-Vázquez et al. 2019). Additionally, they also observed that larvae reared in constant darkness became arrhythmic. In addition, the Clock/Bmal protein complex plays an important role in immunity, directly regulating the transcription of immune-related genes (Sacksteder and Kimmey 2022), which is interesting from a survival point of view. In our study, *clocka* overexpression was observed in the S^+^ group but there were no significant changes in *bmal1b* expression levels (Fig. 3C). Regarding *casp3*, although no significant differences were found in our study (Fig. 3C), other authors have reported the effect of this gene on apoptotic pathways and oxidative stress in zebrafish (Jia et al. 2020; Zhu et al. 2020; Dang et al*. *2023). Caspases are cysteine proteases that cleave their target proteins at specific aspartate residues and play a key role in apoptosis (Bibo-Verdugo and Salvesen 2022). In particular, caspase 3 is responsible for the majority of proteolysis during apoptosis, making it a widely used marker (Asadi et al. 2022). In addition, the stress-related genes *hspa5* and *hspa9* were significantly downregulated in the combined stress condition (Fig. 3C), contrary to expectations, as both are typically upregulated in response to cellular stress. Surprisingly, no changes in expression were detected under the single temperature stressor (Fig. 3A). *hspa9* is a heat shock protein that regulates a wide range of cellular processes, including cell survival, growth and metabolism (Esfahanian et al. 2023). Its downregulation has been associated with the generation of reactive oxygen species (ROS), increased oxidative stress and mitochondrial dysfunction (Priyanka and Seth 2022; Bae et al. 2023). On the other hand, overexpression of *hspa9* provides protection against accumulated proteins, inflammation and neuronal loss, a characteristic feature observed in neurodegeneration (Priyanka and Seth 2022). In our study, the downregulation of *hspa9* observed under stress conditions could indicate a mitochondrial dysfunction that has been associated with neuronal disorders (Park et al. 2014). Such alterations, together with the increase in malformations described above, could be responsible for the observed behavioural changes. Our results also show a negative regulation in *hspa5* (Fig. 3C), a gene that codes for a chaperone that is primarily responsible for the correct folding of proteins in the endoplasmic reticulum (ER). A study by Yang et al. (2020) highlights the effect of negative regulation of *hspa5*, promoting ER stress and generating damage-associated molecular patterns molecules (DAMPs). *hspa5* upregulation increases the proper folding of proteins, so its downregulation increases misfolding of nascent polypeptides, inducing ER stress and triggering the misfolded protein response (UPR) system. Therefore, it is possible that the low levels of *hspa5* observed in our experiment were indicators of ER stress and the presence of misfolded proteins. We also focused on *ddit3,* another gene involved in ER stress. Its overexpression together with the activating transcription factor 4 (ATF4) has been reported to increase protein synthesis, ROS production and deplete ATP, ultimately leading to cell death (Han et al. 2013). However, in our study we did not obtain significant differences in the expression of this candidate gene between the control and the S^+^ group (Fig. 3C).

In the same line we did not register changes in other of the key genes involved in development, *sox2* (Fig. 3C). We explored the expression of this transcription factor since *sox2* KO zebrafish larvae show swim bladder deflation (Cao et al. 2023), similarly to the phenotypes reported in our stressed larvae. We also focused on the retinoic acid (RA) signalling pathway since we reported some ocular abnormalities in some larvae with multiple malformations of the “stressed” (S^+^) group. RA pathway is a key mechanism during embryonic development that can be altered by changes in photoperiod. RA plays a critical role in embryonic development and both excessive and insufficient RA levels cause severe birth defects in the eyes, skull, limbs, brain, heart and other systems and structures (Ross et al. 2000). RA signalling is mediated by nuclear receptors called retinoic acid receptors (RAR) and retinoid receptors (RXR), *rarga* (one of the genes studied in the present work) is a γ RAR (Linville et al. 2009). The key role of RA signalling pathway in early ocular development is supported by previous works (T. Le et al. 2012) in which a pathway chemical inhibitor reduced eye size in zebrafish larvae. However, our gene expression experiments did not show significant differences in *rarga* gene expression (Fig. 3C) between the C and S^+^ groups and therefore we cannot conclude a correlation between this pathway and the observed phenotypes.

We also studied RA since this molecule is also crucial in germ cell development. Primordial germ cells (PGCs) are the embryonic precursors of gametes and migrate within the embryo until they reach the gonadal ridge (Aalto et al. 2021). PGC migration is influenced by the somatic environment and these cells must follow the signals produced by the somatic tissues guiding them towards the gonad where they differentiate into gametes (Raz 2002). The importance of the genes selected in our study in relation to germ cells is supported by numerous scientific articles (Doitsidou et al. 2002; Hartung et al. 2014; Yahiro et al. 2024; Chen et al. 2024): *cxcl12a* and *cxcr4b* are relevant genes for the viability and migration of PGCs, the former is a chemokine expressed in the PGC migration pathway and the latter is a receptor present in the PGC membrane that allows migration towards the domain where the level of expression of *cxcl12a* is higher (Doitsidou et al. 2002; Lau et al. 2020; Yahiro et al. 2024). On the other hand, *ddx4* is one of the main components of germplasm maternally inherited in zebrafish (Schupbach and Wieschaus 1986; Robles et al. 2017). Our immunofluorence results show the presence of a similar number of Ddx4^+^ cells in the C and S^+^ groups (Fig. 4D), which can be correlated with the homogeneous values of Ddx4^+^ transcripts (Fig. 4C). The study of the number of PGCs in early development is interesting from the point of view of gonadal differentiation, as a threshold number of PGCs is required for the stability of the ovary and a depletion of these cells lead to an abortive ovary promoting testis formation (Slanchev et al. 2005; Tzung et al. 2014; Zhou et al. 2018b; Wang et al. 2023). Moreover, alterations in the number or migration of PGCs to the genital ridge can ultimately lead to reproductive problems (Wong and Collodi 2013; Zhou et al. 2018b). Our study also reported a significant decrease in *cxcr4b* expression in the “stressed” (S^+^) group (Fig. 4C). Doitsidou et al. (2002) observed that blocking the transcripts of genes such as *cxcl12a* and *cxcr4b* with an antisense morpholino resulted in the erroneous migration of PGCs during embryonic development. This corresponds with our results, as we observed an abnormal migration pattern in stressed larvae (Fig. 4G), which may be due to the negative regulation found in *cxcr4b*. Despite these interesting findings, additional functional studies are necessary to establish definitive causal connections between transcriptional data and physiological response to stress.

Eventually, we studied tissue regeneration ability in the S^+^ group and their control counterparts to broadly explore the impact of the more aggressive stressful conditions on larvae physiology. Zebrafish, as a teleost, is also a model species for the study of regeneration due to its remarkable ability to regenerate many tissues and organs, including fins (Nakatani et al. 2007; Sehring and Weidinger 2022), heart (Poss et al. 2002), spinal cord (Alper and Dorsky 2022), and optic nerve (Beckers et al. 2023), among others. Among these, the fin is the preferred model for studying regeneration because of its easy access, structural simplicity, and rapid regeneration process. While most regeneration studies have focused on adult tissues, recent research has highlighted that similar cellular and molecular mechanisms occur during fin regeneration in both adult and larval zebrafish (Kawakami et al. 2004; Crossman et al. 2024). Yoshinari and Kawakami reported that all genes induced during larval regeneration were also upregulated during adult fin regeneration, demonstrating comparable gene expression patterns and molecular similarities between both models (Yoshinari and Kawakami 2011). Studying fin regeneration in larval stages, as we did in our study, offers several advantages. The fin regenerates within 3 days post-amputation (dpa), which is much faster than in adult zebrafish and minimizes the impact of environmental factors on regeneration. This makes the larval model particularly useful for studying the effects of stressors on regenerative capacity. Previous studies have shown that chronic stress exposure can disrupt zebrafish fin regeneration (Henríquez Martínez et al. 2022), underlining the importance of evaluating regenerative processes in response to environmental stress, as it provides insights into both the organism’s resilience and the long-term effects of stress on tissue repair mechanisms. Our study showed significant differences in the fin regenerated area between both groups (Fig. 5B). One of the causes of the reduction in the regenerated area in the “stressed” (S^+^) group could be the alteration in the expression levels of regeneration-related genes, such as *hspa9* (Fig. 3C), *cxcr4b* and *cxcl12a* (Fig. 4C), which we have previously discussed. *hspa9* is expressed in blastema, contributing to cell proliferation and tissue growth as a molecular chaperone. Yoshinari et al. (2009) reported that genetic mutations of *hspa9* disrupt the fin regeneration by impairing the blastema cell proliferation, since larval fin of *hspa9* mutants cut at 2 dpf showed a defect in fin regeneration. *cxcr4b* is expressed in the wound epidermis and together with *cxcl12a*, expressed in blastema, plays a critical role in epidermal cell proliferation (Dufourcq and Vriz 2006; Bouzaffour et al. 2009). Neither *cxcr4b* nor *cxcl12a* transcripts are detected in the fin before amputation. However, in regenerating fins these genes show a dynamic expression pattern. Expression of *cxcr4b* is first detected at 1 dpa and *cxcl12a* at 2 dpa, then is rapidly downregulated at 3 dpa and 5 dpa, respectively (Dufourcq and Vriz 2006). This is in line with our results, showing a *hspa9* and *cxcr4b* downregulation (Figs. 3C and 4C) which would explain the reduction in regeneration due to a disruption in blastema and epidermal cell proliferation. The study by Bouzaffour et al. (2009) showed that the expression of *cxcl12a* and *cxcr4b* is regulated during the regeneration process. They found that *cxcr4b* mutant zebrafish regenerates caudal fin normally while *cxcl12a* mutant slows the regeneration process. Despite the intrinsic relationship between *cxcl12a* and *cxcr4b,* our study found no significant differences in the expression of *cxcl12a* (Fig. 4C). Regarding temperature as a stressor, its effect on the fin regenerated length and growth rate has been previously reported (Lee et al. 2005). Similarly, the detrimental effect of stress on regeneration is also supported by a study that reported the disruption in zebrafish fin regeneration caused by the exposure to chronic stress conditions (Henríquez Martínez et al. 2022). These works, together with data presented here, highlight the effect of chronic stress in early stages of the regeneration process which may have also long-term effects on the exposed individuals.

## Conclusion

This study demonstrates that elevated temperature (34 °C), constant darkness (24 h), and their combination act as early-life stressors in zebrafish, inducing distinct yet partially overlapping developmental, behavioural, molecular and regenerative effects. Thermal stress alone promoted early hatching and increased swim bladder malformations without affecting survival. Constant darkness had minimal effects on behaviour and early malformations but reduced hatching at 48 hpf and increased cardiac malformations at 120 hpf. When combined, the stressors impacted survival, increased malformations, reduced fin regeneration and altered larval behaviour.

At the molecular level, the combined stress triggered *clocka* overexpression and downregulated *hspa9* and *hspa5*, while thermal stress alone downregulated *clocka.* Constant darkness had no detectable effect on gene expression. PGC number remained unaffected, but *cxcr4b* was downregulated and PGC migration altered under combined stress.

These results highlight the value of analysing both individual and combined environmental stressors, as their interaction can lead to unique developmental disruptions in zebrafish.

### Supplementary information

## Data Availability

No datasets were generated or analysed during the current study.
